# PRDX1 as a Redox Gatekeeper at Mitochondrial-ER Contact Sites: Implications for Kidney Fibrosis Progression and Therapy

**DOI:** 10.3390/antiox15101263

**Published:** 2026-10-01

**Authors:** Yang Zhang, Jaeyeon Lee, Nan-Nan Yu, Mei-Hua Jin, Ying-Hao Han, Hyo-Jin Park, Deog-Bon Koo, Hu-Nan Sun, Dong-Seok Lee

**Affiliations:** 1 Stem Cell and Regenerative Biology Laboratory, College of Life Science & Biotechnology, Heilongjiang Bayi Agricultural University, Daqing 163319, China; yangzhang@byau.edu.cn (Y.Z.); nannanyu@byau.edu.cn (N.-N.Y.); meihuajin@byau.edu.cn (M.-H.J.); yinghaohan@byau.edu.cn (Y.-H.H.); 2 Heilongjiang Provincial Key Laboratory of Animal Cell Activities and Stress Adaptations, Daqing 163319, China; 3 BK21 FOUR KNU Creative BioResearch Group, School of Life Sciences, Kyungpook National University, Daegu 41566, Republic of Korea; leejydavid@knu.ac.kr; 4 Department of Biotechnology, College of IT Engineering, Daegu University, Gyeongsan 38453, Republic of Koreadbkoo@daegu.ac.kr (D.-B.K.); 5 DU Center for Infertility, Daegu University, Gyeongsan 38453, Republic of Korea; 6 Department of Infertility Medical Industry, College of Health and Biology, Daegu University, Gyeongsan 38453, Republic of Korea

**Keywords:** kidney fibrosis, peroxiredoxins, oxidative stress, reactive oxygen species

## Abstract

Peroxiredoxins (PRDXs) constitute a class of thiol-dependent oxidoreductive regulatory factors whose functions extend beyond peroxide detoxification to include the regulation of oxidoreductive signaling, organelle homeostasis, and cell fate determination. The emerging role of PRDX1–6 in fibrotic diseases—particularly PRDX1’s function as an oxidoreductive signaling modulator dependent on specific pathological contexts during renal injury and fibrosis—calls for further investigation. We propose a dual-regulatory model in which PRDX1 simultaneously governs both the mitochondrial reactive oxygen species (ROS)–JNK/Smad signaling pathway and the endoplasmic reticulum (ER) stress–protein kinase R-like endoplasmic reticulum kinase (PERK)/eukaryotic initiation factor 2α (eIF2α)/activating transcription factor 4 (ATF4)/C/EBP homologous protein (CHOP)-gasdermin E (GSDME) signaling pathway, thereby linking organelle oxidoreductive imbalance to epithelial cell injury, inflammatory cell death, and fibrotic remodeling. Notably, we hypothesize that the mitochondria-associated membranes (MAMs) serve as a critical spatial interface for the convergence of these signaling pathways. At the MAMs, PRDX1 may function as an “oxidoreductive gatekeeper,” buffering local peroxide signaling while coordinating the oxidoreductive balance, Ca^2+^ transport, and mitochondrial homeostasis between the ER and mitochondria. This theoretical framework provides a potential basis for elucidating the context-dependent role of PRDX1 in both acute and chronic renal injury, and identifies the PRDX1–MAMs oxidoreductive gating axis as a promising therapeutic target for renal fibrosis.

## 1. Introduction

Redox homeostasis serves as an essential foundation for maintaining cellular survival and tissue function; peroxiredoxins (PRDXs) represent an important family of intracellular redox-regulating proteins [1,2,3]. In mammals, six members—PRDX1–6—have been identified, all of which possess highly conserved cysteine-dependent peroxidase activity and contribute to the maintenance of cellular redox homeostasis by reducing H_2_O_2_ and other peroxides [1,2,3]. Based on the number and structural characteristics of their catalytic cysteine residues, PRDXs can be classified into three groups: the canonical disulfide-bonded PRDX1–4, the atypical disulfide-bonded PRDX5, and the monosulfide-bonded PRDX6 [4]; each member exhibits distinct subcellular localization, enabling it to function within various cellular compartments—including the cytoplasm, mitochondria, ER, peroxisomes, and the nucleus.

However, simply defining PRDXs as “antioxidant enzymes” is insufficient to explain their complex biological functions. The catalytic cysteine residues in PRDXs not only participate in peroxide scavenging but also undergo reversible oxidative modification, enabling PRDXs to be involved in redox signaling, protein quality control, and cellular stress adaptation [5,6,7]. In other words, PRDXs do not merely passively reduce ROS levels; instead, they can detect changes in the intracellular oxidative state and convert these redox changes into specific signaling outputs [6,7]. Consequently, the function of PRDXs depends on their specific isoform, subcellular localization, oxidative state, and the pathological environment in which the cell is situated. This characteristic also explains why the same PRDXs isoform may exert distinctly different biological effects in different tissues or even at different stages of a disease.

Recent studies have further demonstrated a close relationship between PRDXs and tissue fibrosis. Oxidative stress (OS) is not merely a damaging factor in the fibrotic process; rather, it can contribute to disease progression by modulating inflammatory responses, cell death, the transforming growth factor-β (TGF-β)/Smad and mitogen-activated protein kinase (MAPK) signaling pathways, and other profibrotic signaling mechanisms [8]. In this context, PRDXs can influence cell fate and the tissue microenvironment by regulating local hydrogen peroxide (H_2_O_2_) and other redox signaling pathways, exhibiting distinct context-dependent effects across different organ fibrosis models. For example, PRDX1–5 can exert distinct or even opposing effects in the kidneys, liver, and lungs, suggesting that the biological functions of PRDXs cannot be simply equated to their antioxidant capacity [9].

Among these, PRDX1 holds particularly significant research value. As a widely expressed, disulfide-bonded PRDX isoform, PRDX1 not only scavenges H_2_O_2_ to maintain cellular redox homeostasis but also participates in the regulation of stress signaling through its oxidatively dependent functional switching [9,10]. More notably, the role of PRDX1 exhibits clear environmental dependence: under certain conditions, it exerts a protective effect on cells by limiting ROS accumulation [10]; however, in the context of acute renal injury, PRDX1 can be released as a damage-associated molecular pattern (DAMP), thereby promoting inflammatory responses via the macrophage-inducible C-type lectin (Mincle)/spleen tyrosine kinase (Syk)/nuclear factor-κB (NF-κB) signaling pathway [9]. This seemingly contradictory functional profile suggests that PRDX1 may not merely serve as a ROS “scavenger,” but rather acts as a pivotal redox regulatory hub linking OS, inflammation, and cell fate determination.

Therefore, PRDX should be repositioned as a central node in the fibrotic redox signaling network rather than as a conventional antioxidant enzyme. Particularly during renal fibrosis, PRDX1 may simultaneously integrate mitochondrial OS and ER stress within renal tubular epithelial cells, and influence the fibrotic process through pyroptosis mediated by c-Jun N-terminal kinase (JNK)/Smad and gasdermin E (GSDME) pathways, as well as through macrophage-derived interleukin-6 (IL-6)/Janus kinase (JAK)/signal transducer and activator of transcription 3 (STAT3) paracrine signaling. Furthermore, the mitochondria-associated membranes (MAMs) serve as crucial organelle communication interfaces for redox, Ca^2+^, and metabolic signaling; understanding whether PRDX1 coordinates the functions of mitochondria and the ER by regulating the local redox environment at MAMs could be key to elucidating its renoprotective effects and its dependence on disease stage. Building on this foundation, this study takes the redox regulatory function of PRDXs as a starting point, with a focused examination of the role of PRDX1 in renal fibrosis. It systematically explores the interconnections between PRDX1 and ROS, mitochondrial and ER stress, inflammation, as well as MAMs, and further investigates PRDX1 as a potential therapeutic target bridging redox homeostasis and fibrotic signaling pathways.

## 2. Peroxiredoxins in Organ Fibrosis

### 2.1. Classification and Function of Peroxiredoxin Members

**Figure 1:**
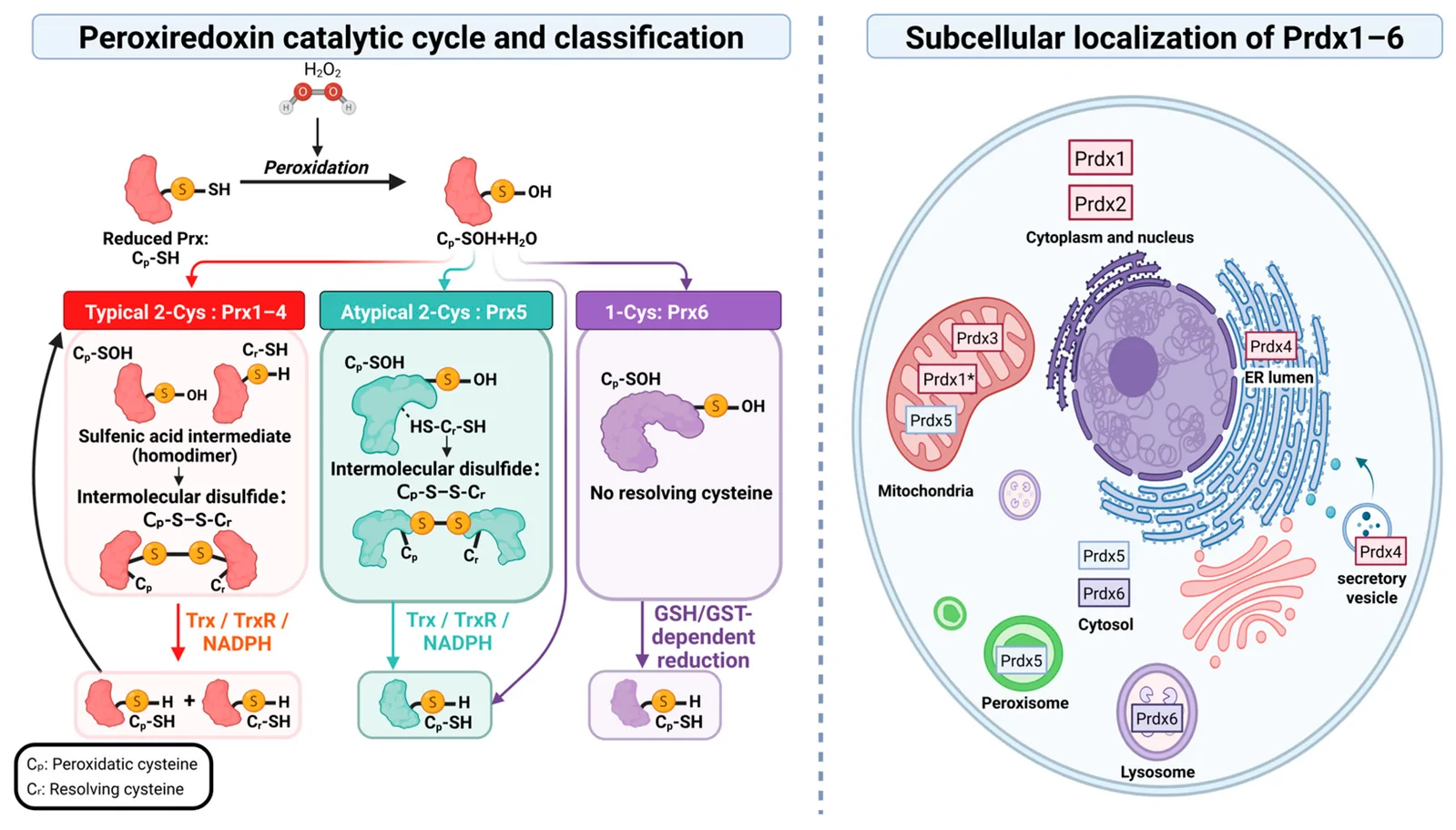
**Catalytic mechanisms and subcellular distribution of the peroxiredoxin family.** PRDX proteins function as thiol-dependent antioxidant enzymes that reduce hydrogen peroxide through reversible cysteine oxidation and reduction cycles. Based on their catalytic mechanisms, PRDXs are divided into typical 2-Cys, atypical 2-Cys, and 1-Cys subgroups. Their diverse subcellular localizations, including cytoplasm, nucleus, mitochondria, ER, and peroxisomes, contribute to compartment-specific maintenance of redox homeostasis. PRDX1* indicates that PRDX1 has been observed in human mitochondria under specific conditions, whereas PRDX3 and PRDX5 are established mitochondrial peroxiredoxins. Created with BioRender.com.

PRDXs constitute a large and highly conserved family of peroxidases that function by reducing peroxides. These enzymes are classified based on the number and location of their conserved redox-sensitive cysteine residues in the catalytic site [1,2,3]. To date, six types of PRDXs have been identified in mammalian cells (Figure 1). The catalytic sites of PRDXs contain cysteine thiol groups (Cys-SH), and these enzymes are categorized into three types: typical disulfide PRDXs (PRDX1–4), atypical disulfide PRDXs (PRDX5), and monosulfide PRDXs (PRDX6) [4]. The conservative thiol peroxidase fold and active site are the functional basis of these enzymes, which primarily regulate signaling pathways through changes in the oxidation state of cysteine, such as the formation of disulfide bonds or peroxides [11,12].

PRDX1 is a typical 2-Cys PRDX with a thioredoxin fold and a conserved catalytic motif [13]. It is widely expressed in various tissues, such as in the kidney, where it is released as DAMPs, exacerbating inflammatory responses [9]. It helps maintain redox homeostasis by scavenging H_2_O_2_ [10]. PRDX2 is another typical 2-Cys PRDX and is one of the primary proteins responsible for scavenging ROS and H_2_O_2_ [14]. In neurons, it regulates ROS-related signaling pathways, protecting cells from oxidative damage [14]. PRDX3 is a mitochondrial-specific H_2_O_2_ scavenger that regulates mitochondrial ROS levels, affecting MMP-1 expression or inhibiting ROS accumulation to indirectly regulate matrix metalloproteinase activity [15]. PRDX4 primarily influences the cellular microenvironment by regulating OS signals in the ER [16]. It can scavenge H_2_O_2_, but in chronic inflammation, it may promote cancer development through ROS signaling [16].

PRDX5 is widely distributed in cells, found in the cytoplasm, mitochondria, peroxisomes, and nucleus, allowing it to exert antioxidant effects in multiple cellular compartments [17,18]. Mitochondrial PRDX5 can scavenge ROS produced by mitochondria, regulate the MAPK signaling pathway, and significantly protect cells from OS [19,20]. Notably, the antioxidant function of PRDX5 is regulated by post-translational modifications [21,22]. For example, α/β hydrolase domain 10 (ABHD10) regulates the palmitoylation state of PRDX5’s active site throughepithelial damage and abnormal tissue repair. Additionally, the oxidation state influences its oligomerization properties [22]. Prdx6, the only 1-Cys Prx, has a unique C-terminal extension structure [23]. It is highly expressed in the lungs and possesses peroxidase, phospholipase A2 (PLA2), and lysophosphatidylcholine acyltransferase (LPCAT) activities [23,24,25]. It can repair oxidized states and act as an antioxidant. It can also generate pro-inflammatory lipids and ROS through PLA2 activity, promoting oxidation and participating in the inflammatory response of respiratory diseases [23,24,25]. Inflammation In Prdx4 and Prdx6 play a role through the ROS–inflammatory–cancer axis [16,24]. Indeed, all Prxs can remove H_2_O_2_ and short-chain hydrogen peroxides, among which Prdx6 can also repair membrane lipid peroxidation [23,26].

### 2.2. Multiorgan Fibrosis: Pathogenic Mechanisms and Therapeutic Frontiers

Cardiac fibrosis is primarily induced by factors such as myocardial infarction, ischemia–reperfusion, mechanical overload, and drug toxicity. In recent years, research has shifted from traditional anti-fibrotic approaches to areas such as immune cell engineering, cell reprogramming, and tissue regeneration. The goal is to reverse cardiac remodeling by regulating the immune microenvironment, inhibiting myofibroblasts, and promoting myocardial regeneration [27,28,29,30,31,32,33,34,35]. Liver fibrosis involves intricate interactions between liver sinusoidal endothelial cells (LSEC) and hepatic stellate cells (HSCs). Epigenetic regulation, RNA modification, lipid metabolism changes, and inflammation all contribute to the sustained activation of HSCs. This area has become a major focus of research in recent years [27,36,37,38,39,40,41,42,43,44]. Pulmonary fibrosis is primarily characterized by alveolar epithelial damage and abnormal tissue repair. Mechanical stimuli, ER stress, and immune cell metabolism all contribute to the formation of the fibrotic microenvironment. In contrast, renal fibrosis is more closely associated with renal tubular epithelial cells. Its development occurs throughout the progression from acute kidney injury (AKI) to chronic kidney disease (CKD). Recent research has led to a new understanding of this process, centered on the sequence of renal tubular epithelial cell injury, immune–inflammatory amplification, and fibroblast activation. Various mechanisms, including ROS-mediated OS, DNA methylation, abnormal complement activation, STING and ER stress, mitochondrial dysfunction, liquid–liquid phase separation, and metabolic reprogramming, interact with each other and ultimately contribute to classic fibrotic pathways such as the TGF-β/Smad pathway, driving abnormal extracellular matrix (ECM) deposition and renal interstitial fibrosis [45,46,47,48,49,50,51,52,53]. Therefore, compared to other organs, renal fibrosis highlights the dynamic interactions between OS, organelle homeostasis, immune inflammation, and metabolic disorders (Table 1) [27,28,29,34,37,39,43,53,54]. In the future, targeting multiple pathogenic pathways rather than individual molecules may become an important strategy for achieving precise anti-fibrotic therapy [45,46,47,48,49,50,51,52,53,55,56,57,58].

### 2.3. The Respective Roles of PRDXs Family Members in Fibrosis of Different Organs

In kidney fibrosis, the roles of PRDXs family members differ significantly from those in other organs such as the liver and lung; their core mechanism focuses on directly or indirectly promoting fibrogenesis by regulating inflammatory responses, OS, and cellular signaling pathways (Table 2) [9,24,56,60,61,62,63,64,65,66,67,68,69,70,71,72,73]. Specifically: PRDX1, upon release as a DAMP molecule, promotes M1 macrophage polarization and pro-inflammatory factor secretion by activating the Mincle/Syk/NF-κB signaling pathway, thereby exacerbating kidney injury. This is distinctly different from its negative regulatory role in fibrosis in the liver (by inducing HSCs apoptosis) and in the lung (by inhibiting EMT and fibroblast proliferation) [9]; In hyperuricemia-induced kidney injury, PRDX2 undergoes S-nitrosylation at Cys172 to form S-nitrosylated PRDX2 (SNO-PRDX2), leading to mitochondrial dysfunction, which in turn aggravates OS and cell apoptosis, ultimately promoting kidney interstitial fibrosis. In contrast, in the liver it primarily inhibits ROS production through antioxidant activity, and in the lung it exerts protective effects as a negative regulator of PDGF signaling [74]; PRDX3 deficiency enhances mitochondrial OS, activates macrophages, and promotes their secretion of pro-inflammatory/pro-fibrotic factors, accelerating fibrogenesis. This contrasts sharply with its anti-fibrotic effects in the liver (by clearing mitochondrial ROS and inhibiting the TGF-β1/Smad pathway) and in the lung (via m6A modification-mediated translational regulation) [75]; PRDX4 inhibits H_2_O_2_ production in the ER. and interleukin-1β (IL-1β)/tumor necrosis factor (TNF) release by interacting with dopamine D5 receptor (D5R) and upregulating its expression via D5R agonist (FEN), thereby alleviating inflammatory injury. This is opposite to its role in the liver (inducing ferroptosis) and in the lung (promoting fibrosis progression and inflammation) [72]; PRDX5 reduces alpha-smooth muscle actin (α-SMA) and ECM deposition by inhibiting the JAK2/STAT3 pathway, delaying fibroblast activation. Its effect in the kidney differs from its role in the liver, where it exacerbates OS and hepatocyte injury [73]. Although PRDXs have been implicated in cardiac fibrosis, the available evidence remains relatively limited compared with that in renal, hepatic, and pulmonary fibrosis [76]. In particular, the specific PRDXs isoforms involved, their cellular and subcellular functions, and the underlying redox-regulatory mechanisms in cardiac fibrosis remain insufficiently characterized. Further studies are therefore warranted to clarify the roles of PRDXs in cardiac fibrogenesis and to determine whether PRDXs-mediated redox regulation represents a potential therapeutic target in cardiac.

## 3. Targeted Translational Strategies Targeting the PRDXs-Related Redox Pathway

### 3.1. Interactive Network Between Oxidative Stress and ER Stress and Mitochondrial Damage

When ROS is excessive, it often damages lipids, proteins and DNA through oxidative injury and destroys cell homeostasis. The activation of nicotinamide adenine dinucleotide phosphate (NADPH) oxidase, such as NADPH oxidase 4 (NOX4) and NADPH oxidase 5 (NOX5), the absence of antioxidant enzymes, such as glutathione peroxidase 3 (GPX3), and mitochondrial dysfunction can all lead to excessive production of ROS [8,77,78]. Among them, the NADPH oxidase family (e.g., NOX4 and NOX5) is the main source of ROS. In diabetic nephropathy, endothelial cell-specific expression of NOX5 directly aggravates kidney fibrosis by activating EMT pathway and ROS signal [77]. The OS microenvironment in the absence of GPX3 can induce the expression of NOX4, which activates fibroblasts through the PKCα/MAPK/STAT3 pathway and drives the progression of fibrosis [8]. Mitochondrial dysfunction, including Dynamin-related protein 1 (DRP1) mediated excessive division, decreased membrane potential and reduced adenosine triphosphate (ATP) synthesis can lead to ROS leakage [79]. These observations indicate that OS not only serves as a damaging factor but also functions as a signaling hub coordinating ER stress and mitochondrial dysfunction.

**Figure 2:**
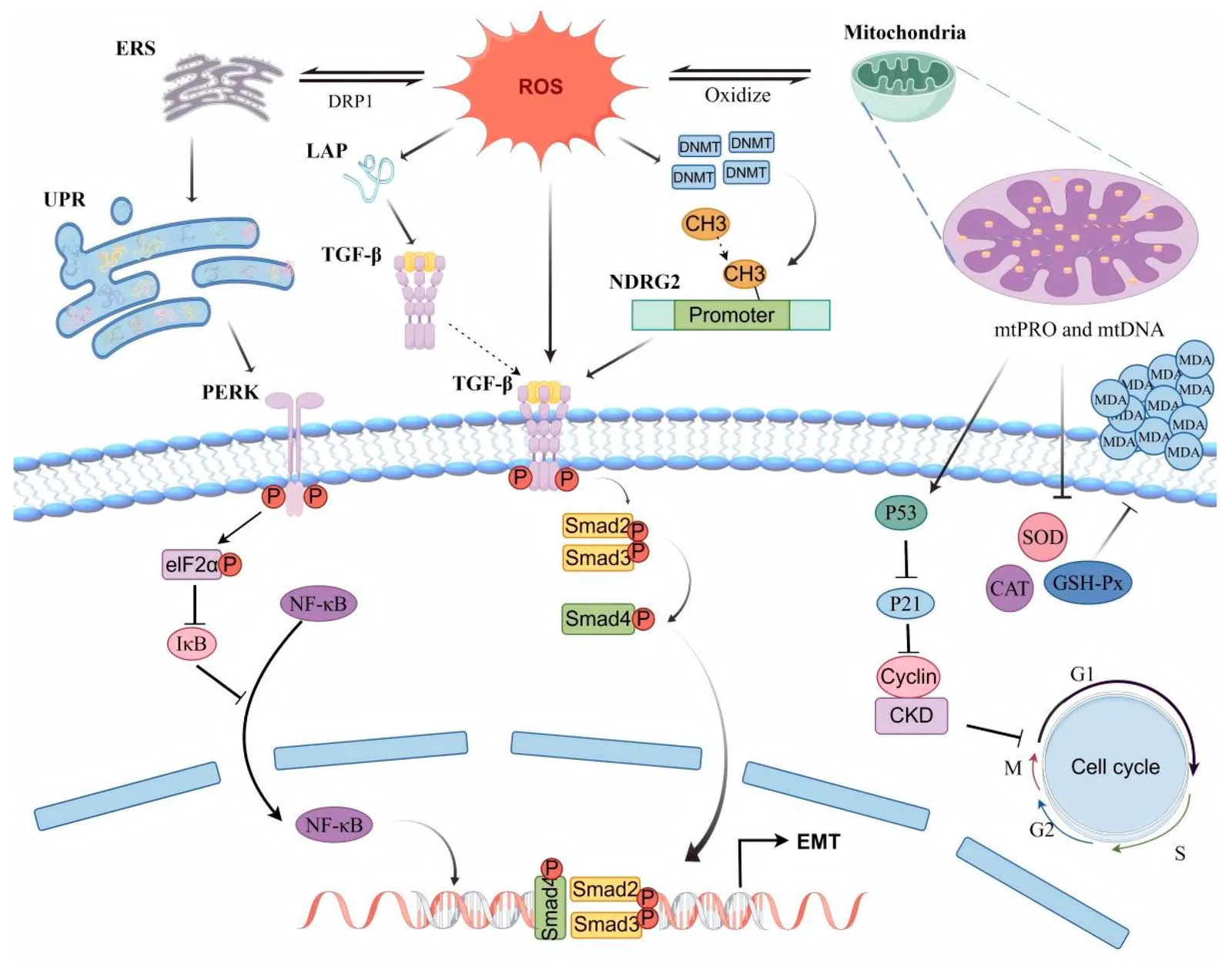
**Mechanistic link between oxidative stress and kidney fibrosis.** ROS accumulation induced by mitochondrial dysfunction and cellular stress activates multiple profibrotic signaling pathways during kidney fibrosis. ROS enhances TGF-β/Smad signaling, promotes **inflammatory** responses through NF-κB activation, and induces cell cycle arrest via the p53/p21 pathway. OS also disrupts antioxidant defense systems such as SOD, CAT, and GSH-Px, thereby amplifying mitochondrial damage and fibrotic signaling. Created with Figdraw.com.

In addition, ROS accumulation acts as an upstream driver that activates NF-κB and TGF-β/Smad signaling, thereby promoting inflammatory responses and ECM deposition [80,81]. For example, oxalic acid can induce pyroptosis of kidney tubular epithelial cells (TECs) through ER stress-ROS-NF-κB pathway, and at the same time activate inflammatory response and accelerate fibrosis [82]. ROS can directly upregulate the expression of TGF-β or activate its latent associated protein by oxidative modification, releasing active TGF-β [83]. ROS also induced hypermethylation of NDRG2 promoter by upregulating DNA methyltransferase, promoted the activation of TGF-β/Smad3 signaling pathway, and inhibited its anti-fibrosis effect [77]. Meanwhile, ER stress activates the PERK-eIF2α pathway through the unfolded protein response (UPR), leading to reduced IκB synthesis, nuclear release of NF-κB, and promotion of inflammation, OS, and fibroblast activation [8,82,84]. Calcium homeostasis imbalance caused by ER stress can also lead to a decrease in mitochondrial membrane potential, promoting the overactivation of mitochondrial division protein DRP1, which in turn intensifies ROS production [82]. ROS can further oxidize mitochondrial proteins and mtDNA, exacerbate kidney damage, and accelerate cell senescence through p53/p21 pathway [85,86,87]. The ROS produced by mitochondrial damage further inhibited the antioxidant enzymes, such as superoxide dismutase (SOD) and glutathione peroxidase (GSH-Px), leading to the accumulation of malondialdehyde (MDA), a lipid peroxidation product, and the continuous amplification of oxidative injury [82]. OS caused by excessive ROS, together with ER stress and mitochondrial damage, forms a vicious cycle of cellular injury during the initiation and progression of kidney fibrosis, thereby exacerbating fibrotic progression through these complex and interactive molecular mechanisms (Figure 2). Currently, the therapeutic efficacy of many antioxidant and stress-modulating therapies remains limited by their inadequate intracellular targeting capability. Emerging therapeutic strategies targeting endogenous antioxidant proteins, epigenetic regulators, and ROS-responsive nanodelivery systems hold promise for enhancing therapeutic precision while minimizing systemic adverse effects [88,89,90]; representative interventions are summarized in Table 3 [88,91,92,93,94,95,96,97,98,99]. Therefore, the precise regulation of renal redox homeostasis—particularly targeted modulation aimed at specific ROS clearance—to mitigate OS could represent a key strategy for halting the fibrotic process.

### 3.2. Future Directions for Understanding PRDX1-Mediated Organelle Redox Homeostasis

**Figure 3:**
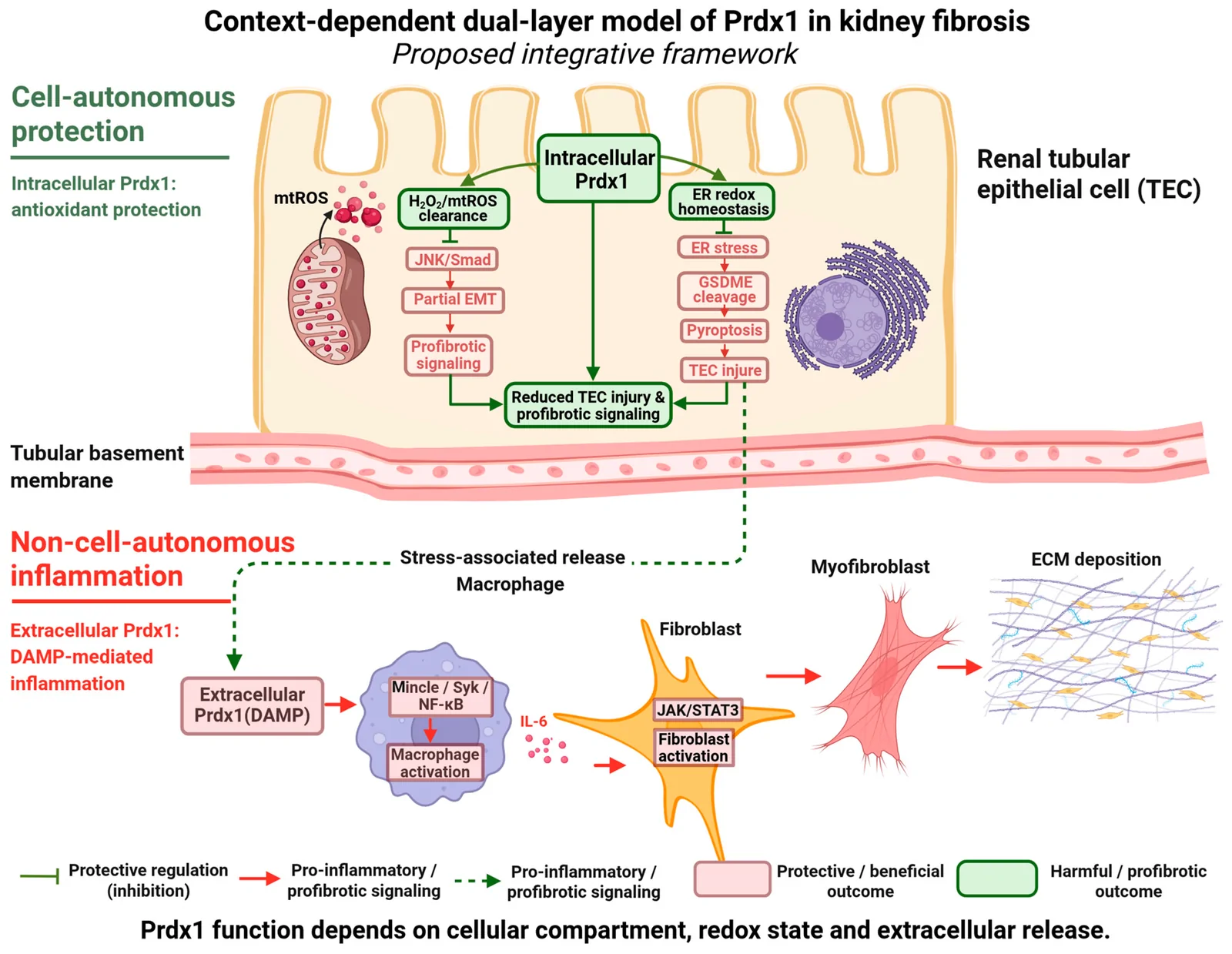
**Context-dependent dual roles of PRDX1 in kidney fibrosis.** PRDX1 exerts distinct effects on renal fibrosis depending on its cellular compartment and redox environment. Intracellular PRDX1 protects tubular epithelial cells by maintaining mitochondrial and endoplasmic reticulum redox homeostasis, thereby limiting mtROS accumulation, JNK/Smad-mediated EMT, ER stress, GSDME-dependent pyroptosis, and profibrotic signaling. In contrast, extracellular PRDX1 may act as a damage-associated molecular pattern to activate macrophage inflammatory responses, IL-6/JAK/STAT3 signaling, fibroblast activation, and extracellular matrix deposition. Created with BioRender.com.

In OS summary, the aforementioned evidence indicates that multiple interconnected pathways collectively contribute to the progression of kidney fibrosis. As previously noted, OS and mitochondrial dysfunction serves as a key driver linking mitochondrial dysfunction with the activation of pro-fibrotic signaling pathways, particularly the TGF-β/Smad and NF-κB pathways, thereby promoting EMT and inflammatory responses [100]. Concurrently, studies suggest that persistent ER stress and UPR signaling pathways lead to tubular epithelial injury and altered cell fate, implying their potential association with regulated cell death pathways [101]. Furthermore, emerging research on PRDXs, particularly PRDX1, reveals their critical roles in maintaining redox homeostasis, regulating mitochondrial reactive oxygen species (mtROS), and influencing the inflammatory microenvironment through macrophage-associated signaling pathways [67]. Despite these advances, most current studies remain isolated in their focus on individual pathways, lacking an integrated framework to elucidate how redox regulation coordinates multiple fibrotic processes at cellular and microenvironmental levels. Therefore, based on the convergence of the aforementioned ROS-related signaling pathways, ER stress responses, and immune-mediated regulation, we propose a dual-axis regulatory model of kidney tubular fibrosis centered on PRDX1. PRDX1 primarily exerts negative regulation on kidney tubular epithelial cell fibrosis through a dual pathway mechanism. At the cellular autonomous level, PRDX1 inhibits the JNK/Smad signaling pathway and blocks the epithelial–mesenchymal transition (EMT) process by mtROS [67]. Simultaneously, it maintains endoplasmic reticulum redox homeostasis, suppresses ER stress-mediated GSDME cleavage, and prevents pyroptosis [102]. At the non-autonomous cellular level, PRDX1 regulates macrophage IL-6 secretion, thereby inhibiting the IL-6/JAK/STAT3 paracrine signaling and mitigating the pro-fibrotic effects of the inflammatory microenvironment on TECs [9]. Overall, these findings support a dual-axis regulatory framework comprising the “PRDX1-mtROS-JNK/Smad-EMT axis” and the “PRDX1-ER stress-GSDME-pyroptosis axis,” which collectively coordinate cellular stress responses and intercellular communication during kidney fibrosis (Figure 3). This integrated model not only provides a mechanistic basis for understanding the multifaceted roles of PRDX1 but also highlights its potential as a therapeutic target, particularly through strategies aimed at preserving or enhancing PRDX1 expression or activity to simultaneously modulate OS, ER stress, and inflammatory signaling pathways in fibrotic nephropathy. Future studies are warranted to experimentally validate this dual-axis model and determine whether enhancing or preserving PRDX1 function can concurrently attenuate the mtROS–JNK/Smad and ER stress–GSDME pyroptosis axes, thereby providing a potential therapeutic strategy for kidney fibrosis.

## 4. Future Perspectives

### 4.1. MAMs in Fibrotic and Renal Pathophysiology: A Potential Redox Signaling Platform

The MAM-specialized regions of close physical and functional interaction between the ER and mitochondria have emerged as critical regulators of organelle communication in kidney diseases [103]. Beyond serving as structural contact sites, MAMs coordinate multiple cellular processes, including Ca^2+^ exchange, lipid metabolism, mitochondrial dynamics, autophagy, and redox signaling, thereby determining mitochondrial adaptation under stress conditions [103].

An increasing body of evidence suggests that MAMs dysfunction is associated with a variety of pathological conditions [104,105,106]. However, the underlying triggering factors and subsequent consequences exhibit strong context-dependent characteristics. In neuropsychiatric disorders, abnormalities in the calcium ion transport mechanism of the inositol 1,4,5-trisphosphate receptor type 3 (IP3R3)–glucose-regulated protein 75 (GRP75)–voltage-dependent anion channel 1 (VDAC1) pathway can lead to MAMs dysfunction, cytosolic stress, mitochondrial damage, and the activation of the NOD-like receptor family pyrin domain-containing 3 (NLRP3) inflammasome [104]. In cardiac and pulmonary fibrosis, the disruption of the mitofusin 2 (MFN2)-dependent MAMs structure impairs mitochondrial metabolism and calcium homeostasis, and promotes fibroblast activation, apoptosis, and extracellular matrix deposition [105,106]. In liver diseases, the remodeling of the MAMs structure is also closely associated with virus–host interactions, lipid metabolism, dysregulation of calcium homeostasis, and inflammatory activation [107,108]. Importantly, a growing body of evidence is beginning to indicate a direct association between MAMs dysfunction and kidney disease. In diabetic nephropathy, damage to the MAMs structure is closely associated with renal dysfunction [109]. Studies have shown that abnormalities in mitochondrial autophagy-related proteins, dyslipidemia, impaired calcium homeostasis, endoplasmic reticulum stress, and mitochondrial dysfunction can all contribute to tubular injury and renal fibrosis. Restoring the function of these related proteins—such as MFN2 or phosphofurin acidic cluster sorting protein 2 (PACS2)—can help mitigate tubular injury and renal fibrosis, as MFN2 has been shown to ameliorate these conditions [110,111]. These findings indicate that mitochondrial autophagy-related proteins are not merely structural connectors, but serve as dynamic regulatory hubs that integrate functions such as calcium ion signaling, mitochondrial metabolism, organelle quality control, and cellular stress responses [110,111]. The latest evidence regarding liver, cardiac, and pulmonary fibrosis further demonstrates that MAMs dysfunction is not limited to specific organs but represents a conserved mechanism that links mitochondrial dysfunction, endoplasmic reticulum stress, dysregulated Ca^2+^ homeostasis, and inflammatory activation to fibrotic remodeling [105,106,107,112]. In renal fibrosis, this function is particularly crucial, as persistent mitochondrial dysfunction and endoplasmic reticulum stress are interconnected through abnormal calcium ion homeostasis and OS, thereby forming a self-perpetuating cycle that leads to tubular injury and extracellular matrix accumulation [113].

**Figure 4:**
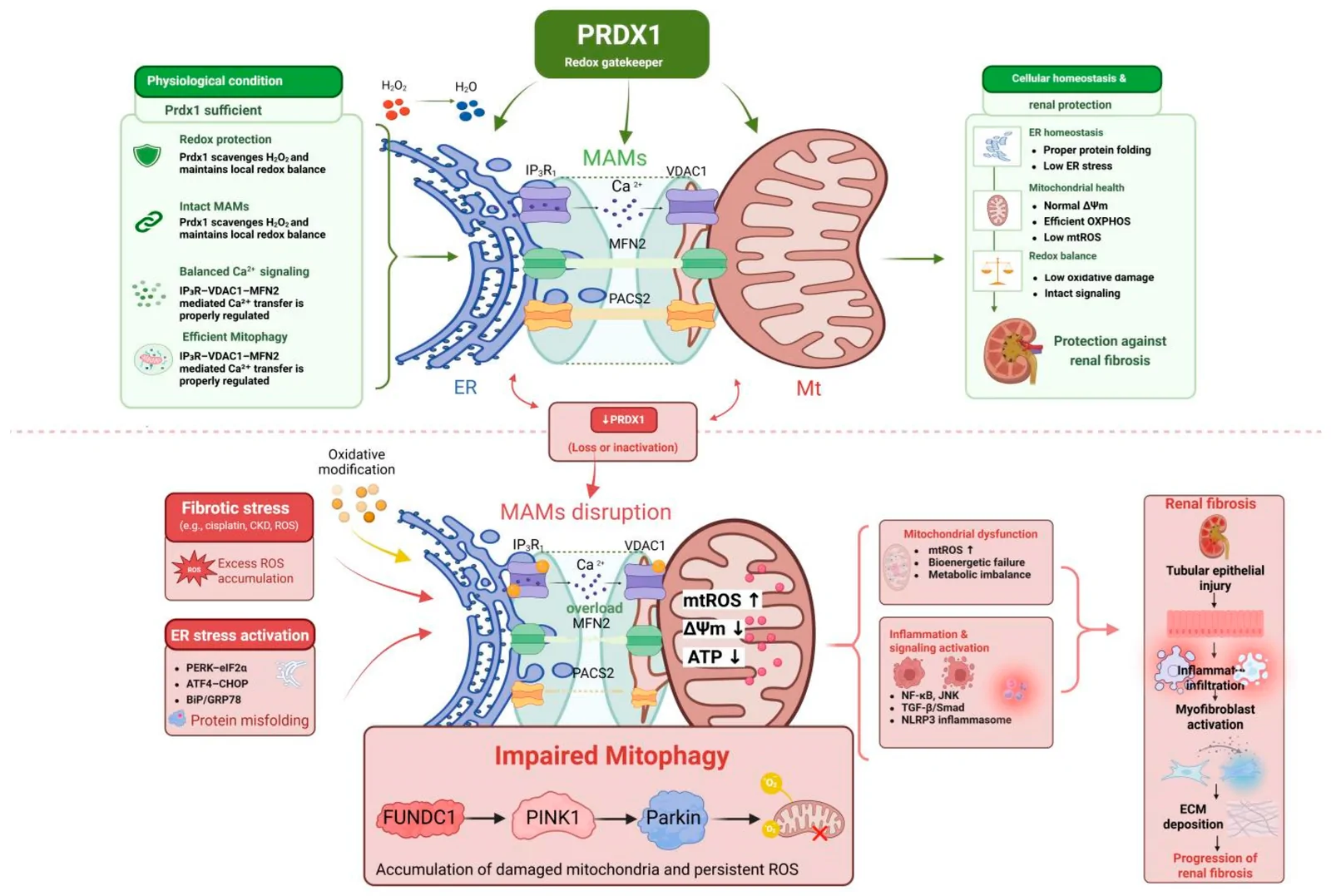
**Potential involvement of PRDX1 in MAMs-associated redox regulation.** Under physiological conditions, PRDX1 may regulate redox homeostasis, calcium ion transport, and mitochondrial function by participating in MAMs-associated redox regulatory processes. Loss or inactivation of PRDX1 induces MAMs disruption, excessive ROS accumulation, ER stress, impaired mitophagy, and mitochondrial dysfunction, characterized by increased mtROS, reduced membrane potential, and decreased ATP production. These alterations promote inflammation, fibroblast activation, and extracellular matrix deposition, ultimately contributing to renal fibrosis progression. Created with BioRender.com. **Abbreviations:** ROS, reactive oxygen species; UPR, unfolded protein response; PERK, protein kinase R-like endoplasmic reticulum kinase; eIF2α, eukaryotic initiation factor 2α; NF-κB, nuclear factor-κB; TGF-β, transforming growth factor-β; ER stress, endoplasmic reticulum stress; CHOP, C/EBP homologous protein; PINK1, PTEN-induced kinase 1; PRDX1, peroxiredoxin 1; mtROS, mitochondrial reactive oxygen species; JNK, c-Jun N-terminal kinase; NLRP3, NOD-like receptor family pyrin domain-containing 3; MAMs, mitochondria-associated membranes; IP_3_R_1_, inositol 1,4,5-trisphosphate receptor 1; VDAC1, voltage-dependent anion channel 1; MFN2, mitofusin 2; PACS2, phosphofurin acidic cluster sorting protein 2; FUNDC1, FUN14 domain-containing protein 1; ATF4, activating transcription factor 4; ΔΨm, mitochondrial membrane potential; ATP, adenosine triphosphate; H_2_O_2_, hydrogen peroxide; Mt, mitochondria.

Recent studies have highlighted that disruption of mitochondria–ER communication, particularly MAMs homeostasis, contributes to renal injury by promoting mitochondrial dysfunction, ER stress, inflammatory activation, and metabolic dysregulation [103,114]. This increasingly evident cross-organ correlation provides a rationale for investigating whether similar MAMs-dependent mechanisms are involved in the process of renal fibrosis. In diabetic kidney disease and other chronic kidney disease models, impaired ER–mitochondria communication has been associated with abnormal Ca^2+^ transfer, mitochondrial ROS accumulation, defective mitochondrial quality control, and progressive renal fibrosis, suggesting that MAMs may represent an important regulatory interface linking organelle dysfunction to fibrotic progression [114]. In renal fibrosis, mitochondrial dysfunction and ER stress have traditionally been considered independent pathological events; however, accumulating evidence indicates that these processes are highly interconnected through dynamic organelle crosstalk [113,115]. Excessive mitochondrial ROS production can disrupt ER protein-folding capacity and calcium homeostasis, whereas persistent ER stress further aggravates mitochondrial injury through abnormal Ca^2+^ flux and OS amplification, forming a self-reinforcing pathological cycle that promotes tubular epithelial injury and extracellular matrix deposition (Figure 4) [113,115].Collectively, these findings position MAMs as a potential regulatory platform connecting ER–mitochondrial dysfunction with renal fibrosis, while the upstream redox regulators that govern this inter-organelle communication remain incompletely understood.

### 4.2. MAMs as a Potential Frontier in PRDX1-Mediated Renoprotection

Despite increasing recognition of MAMs as central regulators of kidney injury, whether antioxidant systems directly control MAMs stability remains poorly understood. PRDX1 represents a potential candidate linking redox regulation with MAMs function. Although traditionally recognized as an H_2_O_2_-scavenging peroxiredoxin, PRDX1 has recently been shown to participate in redox signaling regulation, protein quality control, and stress adaptation through oxidation-dependent functional switching [5,116]. Given that PRDX1 possesses a catalytic activity sensitive to redox conditions and is known to participate in maintaining the homeostasis between mitochondria and the endoplasmic reticulum, PRDX1 is highly likely a candidate protein involved in regulating the local redox environment at the contact site between the endoplasmic reticulum and mitochondria [103,117]. However, whether fibrosis-associated OS alters PRDX1 localization, catalytic activity, or oxidation state, thereby affecting MAMs integrity, remains an important unanswered question.

Several potential mechanisms may explain the involvement of PRDX1 in MAMs regulation. First, excessive ROS accumulation during renal fibrosis may induce oxidative modification of MAMs-associated proteins, leading to abnormal ER–mitochondria tethering and disrupted calcium signaling. Key MAMs components, including IP3R, VDAC1, MFN2, and PACS2, are involved in maintaining organelle coupling and are sensitive to cellular redox changes [118]. By limiting H_2_O_2_ accumulation, PRDX1 may preserve the functional integrity of these protein complexes and prevent excessive mitochondrial Ca^2+^ uptake. Second, PRDX1 may regulate MAMs-dependent mitochondrial quality control. Recent studies have demonstrated that MAMs serve as important platforms coordinating mitophagy-related pathways, including FUN14 domain-containing protein 1 (FUNDC1)-associated and PTEN-induced kinase 1 (PINK1)/Parkin-associated mitochondrial turnover mechanisms [119]. Since defective mitophagy promotes accumulation of damaged mitochondria and persistent ROS production, PRDX1-mediated regulation of MAMs-dependent mitochondrial quality control may represent a previously unexplored mechanism underlying renal protection.

Future studies should therefore determine whether PRDX1 directly localizes to MAMs or indirectly regulates MAMs-associated signaling networks. In diabetic kidney disease, impaired MAMs organization has been implicated in mitochondrial metabolic abnormalities, disturbed Ca^2+^ homeostasis, defective autophagy, and progressive renal injury, suggesting that restoration of ER–mitochondria communication may represent a potential therapeutic strategy for chronic kidney diseases [103,109]. Moreover, emerging evidence indicates that mitochondrial dysfunction and ER stress form a reciprocal pathological network in renal fibrosis, further emphasizing the need to investigate organelle contact sites as regulatory hubs rather than isolated organelle defects [114,120]. Recent advances in proximity-labeling technologies, including TurboID-based interactome mapping, have enabled the identification of transient protein interactions at organelle contact sites and may facilitate the discovery of PRDX1-associated MAMs protein complexes [119]. In parallel, genetically encoded redox sensors targeting mitochondria or the ER provide opportunities to monitor compartment-specific oxidative alterations in real time and may determine whether PRDX1 maintains a protective redox environment at ER–mitochondria interfaces [103]. Combining these approaches with single-cell transcriptomics or spatial multi-omics may further reveal the cell type-specific and stage-dependent roles of the PRDX1–MAMs axis in renal fibrosis. Determining whether PRDX1 directly regulates MAMs integrity or modulates MAMs-dependent signaling may uncover novel mechanisms linking redox regulation, organelle communication, and fibrotic progression.

## 5. Conclusions

In conclusion, MAMs may serve as a critical interface linking the interactions between the endoplasmic reticulum and mitochondria and determining the transition from acute kidney injury to chronic renal fibrosis. By coordinating Ca^2+^ transport, redox balance, endoplasmic reticulum stress, mitochondrial function, and mitochondrial quality control, MAMs can dictate whether acute cellular stress is resolved or progresses into persistent tubular injury and fibrotic remodeling. This framework also helps explain the context-dependent function of 1: while PRDX1 can act as a pro-inflammatory mediator during acute kidney injury, its antioxidant function may be more critical in the context of chronic injury, thereby aiding in maintaining the homeostasis between the endoplasmic reticulum and mitochondria and limiting the progression of fibrosis. However, whether MAMs indeed provide a mechanistic basis for this functional transition remains unclear. Future studies should aim to elucidate the dynamic changes in PRDX1 localization across different stages of disease, as well as the trends in its oxidation state. Emerging evidence suggests that MAMs-mediated interactions between the nucleolus and mitochondria may contribute to the progression from acute kidney injury to chronic kidney disease, exerting its influence by regulating calcium homeostasis, redox signaling, nucleolar stress, and mitochondrial function [121,122]. Addressing this question could facilitate the identification of therapeutic targets for the PRDX1–MAMs axis in renal fibrosis, thereby delineating therapeutic windows at different disease stages and in specific organs.

## Figures and Tables

**Table 1:** **Integrated mechanisms and therapeutic strategies involved in multi-organ.** “↑” indicates significant upregulation, whereas “↓” indicates significant downregulation compared with the corresponding model group. Representative fold changes reported in the original studies are shown. Fold-change values and statistical analyses were derived from the respective references; differences in experimental conditions and normalization methods should be considered when comparing values across studies.

Organ	Etiology	Molecular Mechanism	Metabolic Pathway	Inflammation	Relevant Targeted Drugs	Reference
Cardiac	Myocardial infarction	MI → PHF7↑ → chromatin remodeling (CTCF accessibility↑, Jun/Fos/AP-1 activity↓) → fibroblast-to-cardiomyocyte reprogramming → activated myofibroblasts (Postn, Nox4, Meox1)↓ → cardiac fibrosis & improved cardiac function↓	Epigenetic regulation	Reduced myofibroblast activation	PHF7 gene therapy	[27]
	Myocardial infarction	MI → Nppa/pro-ANP↓ → ANP/NPR1/cGMP-PKG signaling↓ → impaired regenerative paracrine signaling → Postn^+^ fibroblast expansion & ECM deposition → cardiac fibrosis	ANP/NPR1/cGMP-PKG signaling	IL-6/TNF-α-associated inflammation	ANP replacement; NPR1 agonists	[28]
	Myocardial infarction	MI → FAP^+^ fibroblasts recruit FAP-CAR Tregs → IL-10↑ → TGF-β/Smad2/3↓ → myofibroblast differentiation↓ → cardiac fibrosis↓	IL-10/TGF-β/Smad2/3 signaling	Reduced inflammatory cytokines	FAP-CAR Treg therapy	[29]
	Doxorubicin-induced cardiomyopa	Dox → IL-1β/NF-κB → CD47 (“Don’t eat me” signal)↑ → resident macrophage efferocytosis↓ → fibroblast clearance↓ + IGF-1 secretion↓ → POSTN^+^ activated fibroblasts↑ → ECM deposition → cardiac fibrosis	Glutathione metabolism; ferroptosis	IL-1β/NF-κB inflammatory	CD47-SIRPα blockade	[34]
Liver	Chronic liver injury; CCl_4_; DDC	BRD4-dependent SE → PML → PML/BRD4 LLPS → TIMP1/CCL2/CXCL1/IL-1β↑ → TIMP1^+^ LSEC recruits CD63^+^ MoMF → HSCs activation → fibrosis	Epigenetic regulation; LLPS	CCL2/CXCL1-mediated immune recruitment	BRD4 inhibitors; PML inhibition	[36]
	MASH; MetALD	JUNB → LARP6↑ → binds collagen (COL1A1/COL1A2/COL3A1) mRNAs → enhances collagen translation & secretion → HSCs activation → ECM deposition → fibrosis	RNA regulation; collagen synthesis	Profibrotic inflammatory signaling	LARP6 inhibitors	[37]
	MASH; MASLD; CCl_4_	NFIB↓ → CCL5↑ → ROS↑ → HSCs activation → collagen I/III & α-SMA↑ → ECM deposition → fibrosis; NFIB represses CCL5 transcription	OS; ROS metabolism; NADPH oxidase;	CCL5-mediated infiltration;	CCL5 inhibitors; NFIB activation	[39]
	Metabolic dysfunction; MASLD	Excess FA uptake + DNL↑ + mitochondrial FAO↓ → lipotoxic metabolites → hepatocyte injury → macrophage activation + HSCs activation → ECM deposition → MASH fibrosis	Lipid metabolism; mitochondrial dysfunction	NLRP3/NF-κB inflammation	THRβ; GLP-1/GIP; FGF21 agonists	[43]
Pulmonary	IPF; PPF	LPA → LPA1 → epithelial apoptosis → fibroblast recruitment → myofibroblast differentiation → ECM deposition → pulmonary fibrosis	LPA/LPA1 signaling	Profibrotic inflammatory microenvironment	LPA1 antagonist; Nintedanib; Pirfenidone	[54]
	Lung injury; pulmonary fibrosis	Lymphatic dysfunction → IL-1β/IL-1R1–HAS1 activation → hyaluronan accumulation → fibroblast activation → ECM deposition → pulmonary fibrosis	Hyaluronan metabolism; ECM remodeling	IL-1β/IL-1R1 signaling	HAS inhibitors; IL-1R1 blockade	[56]
	Bleomycin-induced lung injury; IPF	eATP → P2RX4 activation in fibroblasts → IL-6 secretion → myeloid ARG1 induction → ornithine-derived proline synthesis → collagen production → pulmonary fibrosis	Arginine-proline metabolism	IL-6-mediated fibroblast activation	ARG1 inhibitors; P2RX4 inhibitors	[57]
		TRPML1↓ → impaired lysosomal exocytosis → MMP2/9/12/19 secretion↓ → reduced ECM degradation → ECM accumulation → pulmonary fibrosis	ER stress/UPR	Epithelial injury-associated inflammation	IRE1α inhibitors	[58]
Kidney	AKI-to-CKD; IRI; UUO; AAN	Failed-repair PTCs → C3/CFB↑ → C3aR/C5aR1 activation → NF-κB/NLRP3/TGF-β → macrophage recruitment → myofibroblast activation → ECM deposition → fibrosis	Complement pathway; mitochondrial dysfunction	Macrophage/neutrophil infiltration	C3/C5aR inhibitors	[45]
	CKD	ROS → NDRG2 promoter hypermethylation → NDRG2↓ → TGF-β/Smad → ECM deposition → renal fibrosis	ROS metabolism; DNA methylation	OS-mediated inflammation	Mito-TEMPO;	[48]
	UUO; CKD	CHOP → EVA1A → BIP–TGFBR2 → TGF-β/SMAD2/3 → ECM deposition → renal fibrosis	ER stress/UPR; TGF-β/SMAD signaling	ER stress-associated inflammation	EVA1A inhibition; TGF-β inhibitors	[49]
	UUO; CKD	STING → PERK activation → ATF4/CHOP-mediated ER stress → tubular injury → inflammatory cytokine release → ECM deposition → renal fibrosis	cGAS–STING signaling; ER stress	IFN-I/CCL2/IL-6 signaling	STING inhibition; PERK inhibition	[51]

**Abbreviations:** AKI, acute kidney injury; ANP, atrial natriuretic peptide; AP-1, activator protein 1; AAN, aristolochic acid nephropathy; ARG1, arginase 1; ATF4, activating transcription factor 4; BIP, binding immunoglobulin protein; BRD4, bromodomain-containing protein 4; CAR, chimeric antigen receptor; C3, complement component 3; C3aR, complement component 3a receptor; C5aR1, complement component 5a receptor 1; CCl_4_, carbon tetrachloride; CCL2, C-C motif chemokine ligand 2; CCL5, C-C motif chemokine ligand 5; cGAS, cyclic GMP-AMP synthase; cGMP, cyclic guanosine monophosphate; CHOP, C/EBP homologous protein; CKD, chronic kidney disease; COL1A1, collagen type I alpha 1 chain; COL1A2, collagen type I alpha 2 chain; COL3A1, collagen type III alpha 1 chain; CTCF, CCCTC-binding factor; CXCL1, C-X-C motif chemokine ligand 1; DDC, 3,5-diethoxycarbonyl-1,4-dihydrocollidine; DNL, de novo lipogenesis; Dox, doxorubicin; ECM, extracellular matrix; eATP, extracellular ATP; ER, endoplasmic reticulum; EVA1A, eva-1 homolog A; FA, fatty acid; FAP, fibroblast activation protein; FAO, fatty acid oxidation; FGF21, fibroblast growth factor 21; GLP-1, glucagon-like peptide-1; GIP, glucose-dependent insulinotropic polypeptide; HAS1, hyaluronan synthase 1; HDG, hederagenin; HK2, hexokinase 2; HSCs, hepatic stellate cells; IFN-I, type I interferon; IGF-1, insulin-like growth factor 1; IL-1β, interleukin-1 beta; IL-1R1, interleukin-1 receptor 1; IL-6, interleukin-6; IPF, idiopathic pulmonary fibrosis; IRE1α, inositol-requiring enzyme 1 alpha; IRI, ischemia–reperfusion injury; JUNB, JunB proto-oncogene; LARP6, La-related protein 6; LPA, lysophosphatidic acid; LPA1, lysophosphatidic acid receptor 1; LSEC, liver sinusoidal endothelial cell; LLPS, liquid–liquid phase separation; MASLD, metabolic dysfunction-associated steatotic liver disease; MASH, metabolic dysfunction-associated steatohepatitis; MetALD, metabolic dysfunction-associated steatotic liver disease with increased alcohol intake; MI, myocardial infarction; Mito-TEMPO, mitochondria-targeted superoxide dismutase mimetic; MMP, matrix metalloproteinase; MoMF, monocyte-derived macrophage; NFIB, nuclear factor I B; NF-κB, nuclear factor kappa B; NDRG2, N-myc downstream-regulated gene 2; NLRP3, NLR family pyrin domain-containing 3; NOX4, NADPH oxidase 4; NPR1, natriuretic peptide receptor 1; Nppa, natriuretic peptide A; OS, oxidative stress; P2RX4, P2X purinergic receptor 4; PERK, protein kinase R-like endoplasmic reticulum kinase; PHF7, PHD finger protein 7; PKG, protein kinase G; PML, promyelocytic leukemia protein; PPF, progressive pulmonary fibrosis; PTCs, proximal tubular cells; ROS, reactive oxygen species; SE, super-enhancer; SIRPα, signal regulatory protein alpha; Sost, sclerostin; STING, stimulator of interferon genes; TGF-β, transforming growth factor beta; TGFBR2, transforming growth factor beta receptor 2; THRβ, thyroid hormone receptor beta; TIMP1, tissue inhibitor of metalloproteinases 1; TNF-α, tumor necrosis factor alpha; TRPML1, transient receptor potential mucolipin 1; Tregs, regulatory T cells; UPR, unfolded protein response; UUO, unilateral ureteral obstruction [27,28,29,34,37,39,43,54,56,57,59].

**Table 2:** **Roles of PRDXs family members in organ fibrosis: localization, mechanisms, and biological outcomes.** “↑” indicates upregulation, whereas “↓” indicates downregulation compared with the corresponding model group. Changes in PRDX expression and related molecular markers are summarized based on findings reported in the original studies. Experimental conditions, detection methods, and statistical analyses varied among studies; therefore, please refer to the original references for detailed information.

Organ	PRXS Family Member	Regulatory Mechanism	Core Effect	References
Liver	PRDX1/ PRDX2	Covalent binding of 18β-glycyrrhetinic acid (18b-GA) inhibits antioxidant activity	ROS induces HSCs apoptosis↑, attenuates fibrosis	[60,61]
	PRDX3	m6A modification regulates translation via YTHDF3	Inhibits mitochondrial ROS/TGF-β1/Smad pathway, suppresses HSCs activation	[62]
	PRDX4	Celastrol binding inhibits activity and upregulates HO-1	Induces ferroptosis ROS/lipid peroxidation iron accumulation)↑	[60]
	PRDX5	ABHD10 deficiency causes S-palmitoylation-induced inactivation	Aggravates OS and hepatocyte damage	[63]
		Overexpression suppresses cytosolic/ mitochondrial ROS and lipogenic genes	Reduces hepatic lipid accumulation, ameliorates steatosis	[64]
	PRDX6	Mutually promoted secretion with MANF (enzyme-independent)	Inhibits NF-κB pathway, downregulates α-SMA/collagen	[60,65]
		Miz1 degradation → PRDX6 release → binds Parkin-Cys431	Inhibits Parkin autoubiquitination → blocks mitophagy → mitochondrial damage → TNFα release	[66]
Pulmonary	PRDX1	Knockdown increases ROS, activates PI3K/Akt and JNK/Smad pathways	Negatively regulates fibrosis by inhibiting EMT and fibroblast proliferation; loss exacerbates fibrosis	[67]
	PRDX2	Negative regulator of PDGF signaling; Loss enhances H_2_O_2_ production and PDGFR activation	Antioxidant defense against H_2_O_2_; Redox signaling regulation; Cell proliferation control	[68]
	PRDX3	Downregulated in fibrosis; m6A modification mediated by YTHDF3	Anti-fibrotic: Clears mitochondrial ROS, inhibits TGF-β1/Smad pathway, suppresses HSCs activation	[62]
	PRDX4	Overexpression in transgenic mice; oxidation-induced oligomerization (HMW formation)	Promotes fibrosis progression and inflammation; serum levels correlate with disease severity	[69,70]
	PRDX5	---	---	---
	PRDX6	Binding of GHK-Cu inhibits OS	Reduces ROS, suppresses inflammation and fibrosis by alleviating alveolar macrophage damage	[71]
		Regulated by ROS, Nrf2, and microRNAs; dual peroxidase and PLA2 activities	Dual roles: antioxidant (scavenges peroxides) and pro-oxidant (activates NOX2); context-dependent effects	[24]
Kidney	PRDX1	Released as a DAMP molecule	Activates Mincle/Syk/NF-κB signaling → Pro-inflammatory factors/M1 macrophage polarization↑ → Aggravates kidney injury	[9]
	PRDX2	S-nitrosylation at Cys172 (SNO-PRDX2)	Mitochondrial dysfunction → OS/apoptosis↑ → Promotes kidney interstitial fibrosis	[74]
	PRDX3	Knockout-induced loss of function	Mitochondrial OS↑ → Macrophage activation → Secretion of pro-inflammatory/pro-fibrotic factors↑ → Accelerates fibrosis	[75]
	PRDX4	Interacts with D5R; D5R agonist (FEN) upregulates expression	Inhibits ER H_2_O_2_ generation → IL-1β/TNF and ER stress↓ → Attenuates inflammatory injury	[72]
	PRDX5	Downreg Shenkang (SK)	Inhibits JAK2/STAT3 pathway → α-SMA/ECM deposition↓ → Delays fibroblast activation	[73]
	PRDX6	Binds AE1 via Cys47	Stabilizes AE1 protein → Maintains acid–base balance → Reduces proximal tubule vacuolization	[56]

**Abbreviations:** PRDXs, peroxiredoxins; 18β-GA, 18β-glycyrrhetinic acid; ROS, reactive oxygen species; HSCs, hepatic stellate cells; m6A, N6-methyladenosine; YTHDF3, YTH N6-methyladenosine RNA binding protein 3; HMW, high molecular weight; OS, oxidative stress; MANF, mesencephalic astrocyte-derived neurotrophic factor; NF-κB, nuclear factor kappa B; PLA2, phospholipase A2; Nrf2, nuclear factor erythroid 2-related factor 2; NOX2, NADPH oxidase 2; EMT, epithelial–mesenchymal transition; PDGF, platelet-derived growth factor; PDGFR, platelet-derived growth factor receptor; PI3K, phosphoinositide 3-kinase; JNK, c-Jun N-terminal kinase; DAMP, damage-associated molecular pattern; Mincle, macrophage-inducible C-type lectin; Syk, spleen-associated tyrosine kinase; SNO, S-nitrosylation; D5R, dopamine D5 receptor; FEN, fenoldopam; ER, endoplasmic reticulum; IL-1β, interleukin-1 beta; TNF-α, tumor necrosis factor alpha; JAK2, Janus kinase 2; STAT3, signal transducer and activator of transcription 3; ECM, extracellular matrix; AE1, anion exchanger 1; GHK-Cu, glycyl-L-histidyl-L-lysine-copper; SK, Shenkang; TGF-β1, transforming growth factor beta 1; ABHD10, abhydrolase domain-containing 10; Miz1, Myc-interacting zinc-finger protein 1; Parkin, parkin RBR E3 ubiquitin protein ligase.

**Table 3:** **Targeted therapeutic agents for organ-specific fibrosis and their mechanisms.**

Organ	Therapeutic Agent	Mechanisms	Reference
Liver	Celastrol	(1) Inhibit PRDX1, PRDX2, PRDX4, and PRDX6, suppress antioxidant activity	[60]
		(2) Promotes ferroptosis in HSCs	
	18β-glycyrrhetinic	(1) Targeted inhibition of PRDX1 and PRDX2 activity	[61]
		(2) Increase ROS levels, induce apoptosis of activated HSCs	
Pulmonary	ISO-1	Inhibit MIF suppresses the negative feedback effect of the PRDX1-MIF axis	[91]
Kidney	3,5,6,7,8,3′,4′-heptamethoxyflavone	(1) Inhibit NEU4’s role in promoting YAP protein nuclear translocation (2) Inhibit NEU4’s activation of fibrotic gene expression	[92]
	DWN12088	Inhibit fibroblast activation and proximal tubule injury	[93]
	Bemcetinib	Increase AXL expression in multiple cell types	[94]
	Raspberry	Inhibit the PI3K/AKT pathway alleviates kidney fibrosis and inflammation	[95]
	hiPSC-NPCs	Secreting vascular endothelial growth factor (VEGF-A) alleviates kidney fibrosis and aging	[96]
	GSK-126	Inhibit EZH2-mediated Smad3 dephosphorylation via DUSP23 enhances the MMT process	[97]
	Ceapin 7	(1) Regulate M2c phenotype macrophage polarization	[88]
		(2) Inhibit TGF-β/Smad3 signaling	
		(3) prevent the excessive polarization of MMT	
	dexamethasone	(1) Regulate M2c phenotype macrophage polarization	[88]
		(2) Inhibit TGF-β/Smad3 signaling	
		(3) prevent the excessive polarization of MMT	
	4-PBA	Inhibit the PERK-PKCδ pathway	[89]
	L-theanine	Inhibit NOX4 thereby suppressing the ROS-TGF-β/Smad3 signaling axis	[90]
	Mito-TEMPO	Inhibit DNMT expression, reduce NDRG2 promoter methylation	[77]
	5-AZA-dC	(1) Inhibit DNMT methylation (2) Reverse NDRG2 expression (3) Block the TGF-β/Smad3 signaling pathway	[77]
	α-tocopherol	Antioxidant, maintain normal mitochondrial energy metabolism	[98]
	C-Mn_3_O_4_ NPs	(1) Simulate SOD/CAT enzyme activity to clear ROS	[99]
		(2) Regulate the activity of immune cells	

**Abbreviations:** PRDXs, peroxiredoxin; ROS, reactive oxygen species; HSCs, hepatic stellate cells; MIF, macrophage migration inhibitory factor; NEU4, neuraminidase 4; YAP, Yes-associated protein; AXL, AXL receptor tyrosine kinase; PI3K/AKT, phosphoinositide 3-kinase/protein kinase B; hiPSC-NPCs, human induced pluripotent stem cell-derived nephron progenitor cells; VEGF-A, vascular endothelial growth factor A; EZH2, enhancer of zeste homolog 2; DUSP23, dual specificity phosphatase 23; MMT, macrophage-to-myofibroblast transition; TGF-β, transforming growth factor beta; Smad3, SMAD family member 3; PERK, protein kinase R-like endoplasmic reticulum kinase; PKCδ, protein kinase C delta; DNMTs, DNA methyltransferases; NDRG2, N-myc downstream-regulated gene 2; 4-PBA, 4-phenylbutyric acid; 5-AZA-dC, 5-aza-2′-deoxycytidine; NPCs, nephron progenitor cells; TEMPO, 2,2,6,6-tetramethylpiperidine-1-oxyl; NPs, nanoparticles; SOD, superoxide dismutase; CAT, catalase.

## Data Availability

No new data were created or analyzed in this study. Data sharing is not applicable to this article.

## Abbreviations

AAN, aristolochic acid nephropathy; ABHD10, abhydrolase domain-containing 10; AE1, anion exchanger 1; AKI, acute kidney injury; ALD, alcohol-associated liver disease; ANP, atrial natriuretic peptide; AP-1, activator protein 1; ARG1, arginase 1; ATF4, activating transcription factor 4; ATF6, activating transcription factor 6; ATP, adenosine triphosphate; AXL, AXL receptor tyrosine kinase; BIP, binding immunoglobulin protein; BRD4, bromodomain-containing protein 4†; C3, complement component 3; C3aR, complement component 3a receptor; C5aR1, complement component 5a receptor 1; CAR, chimeric antigen receptor; CAT, catalase; CCl_4_, carbon tetrachloride; CCL2, C-C motif chemokine ligand 2; CCL5, C-C motif chemokine ligand 5; cGAS, cyclic GMP-AMP synthase; cGMP, cyclic guanosine monophosphate; CHOP, C/EBP homologous protein; CKD, chronic kidney disease; COL1A1, collagen type I alpha 1 chain; COL1A2, collagen type I alpha 2 chain; COL3A1, collagen type III alpha 1 chain; CTCF, CCCTC-binding factor; CXCL1, C-X-C motif chemokine ligand 1; D5R, dopamine D5 receptor; DAMP, damage-associated molecular pattern; DDC, 3,5-diethoxycarbonyl-1,4-dihydrocollidine; DNMT, DNA methyltransferase; DNMTs, DNA methyltransferases; DNL, de novo lipogenesis; Dox, doxorubicin; DR5, death receptor 5; DRP1, dynamin-related protein 1; DUSP23, dual specificity phosphatase 23; ECM, extracellular matrix; eATP, extracellular ATP; eIF2α, eukaryotic initiation factor 2α; EMT, epithelial–mesenchymal transition; ER, endoplasmic reticulum; ERAD, endoplasmic reticulum-associated degradation; EVA1A, eva-1 homolog A; FA, fatty acid; FAP, fibroblast activation protein; FAO, fatty acid oxidation; FEN, fenoldopam; FGF21, fibroblast growth factor 21; FN, fibronectin; GHK-Cu, glycyl-L-histidyl-L-lysine-copper; GIP, glucose-dependent insulinotropic polypeptide; GLP-1, glucagon-like peptide-1; GPX3, glutathione peroxidase 3; GSDME, gasdermin E; GSH-Px, glutathione peroxidase; HAS1, hyaluronan synthase 1; HDG, hederagenin†; HK2, hexokinase 2; HMW, high molecular weight; HO-1, heme oxygenase-1; HSC, hepatic stellate cell; HSCs, hepatic stellate cells; IFN-I, type I interferon; IGF-1, insulin-like growth factor 1; IL, interleukin; IL-1β, interleukin-1 beta; IL-1R1, interleukin-1 receptor 1; IL-6, interleukin-6; IP3R, inositol 1,4,5-trisphosphate receptor; IP3R3, inositol 1,4,5-trisphosphate receptor type 3; IPF, idiopathic pulmonary fibrosis; IRE1α, inositol-requiring enzyme 1 alpha; IRI, ischemia–reperfusion injury; JAK, Janus kinase; JAK2, Janus kinase 2; JNK, c-Jun N-terminal kinase; JUNB, JunB proto-oncogene; LARP6, La-related protein 6; LPA, lysophosphatidic acid; LPA1, lysophosphatidic acid receptor 1; LSEC, liver sinusoidal endothelial cell; LLPS, liquid–liquid phase separation; LPCAT, lysophosphatidylcholine acyltransferase; MANF, mesencephalic astrocyte-derived neurotrophic factor; MAMs, mitochondria-associated membranes; MAPK, mitogen-activated protein kinase; MASLD, metabolic dysfunction-associated steatotic liver disease; MASH, metabolic dysfunction-associated steatohepatitis; m6A, N6-methyladenosine; MetALD, metabolic dysfunction-associated steatotic liver disease with increased alcohol intake; MFN2, mitofusin 2; MI, myocardial infarction; MIF, macrophage migration inhibitory factor; Mincle, macrophage-inducible C-type lectin; Mito-TEMPO, mitochondria-targeted superoxide dismutase mimetic; Miz1, Myc-interacting zinc-finger protein 1; MMP, matrix metalloproteinase; MMT, macrophage-to-myofibroblast transition; MoMF, monocyte-derived macrophage; mtDNA, mitochondrial DNA; mtRNA, mitochondrial RNA; mtROS, mitochondrial reactive oxygen species; NAC, N-acetylcysteine; NDRG2, N-myc downstream-regulated gene 2; NF-κB, nuclear factor kappa B; NFIB, nuclear factor I B; NLRP3, NLR family pyrin domain-containing 3; NOX, NADPH oxidase; NOX2, NADPH oxidase 2; NOX4, NADPH oxidase 4; NOX5, NADPH oxidase 5; NPR1, natriuretic peptide receptor 1; Nppa, natriuretic peptide A; Nrf2, nuclear factor erythroid 2-related factor 2; NPs, nanoparticles; NPCs, nephron progenitor cells; OS, oxidative stress; P2RX4, P2X purinergic receptor 4; PACS2, phosphofurin acidic cluster sorting protein 2; Parkin, parkin RBR E3 ubiquitin protein ligase; PDGF, platelet-derived growth factor; PDGFR, platelet-derived growth factor receptor; PERK, protein kinase R-like endoplasmic reticulum kinase; PHF7, PHD finger protein 7; PI3K, phosphoinositide 3-kinase; PINK1, PTEN-induced kinase 1; PKCα, protein kinase C alpha; PKCδ, protein kinase C delta; PKG, protein kinase G; PML, promyelocytic leukemia protein; PPF, progressive pulmonary fibrosis; PRDX, peroxiredoxin; PRDXs, peroxiredoxins; PTCs, proximal tubular cells; ROS, reactive oxygen species; SE, super-enhancer; SIRPα, signal regulatory protein alpha; SNO, S-nitrosylation; SOD, superoxide dismutase; Sost, sclerostin; SPP1, secreted phosphoprotein 1; STAT3, signal transducer and activator of transcription 3; STING, stimulator of interferon genes; Syk, spleen-associated tyrosine kinase; TGF-β, transforming growth factor beta; TGF-β1, transforming growth factor beta 1; TGFBR2, transforming growth factor beta receptor 2; TECs, tubular epithelial cells; TEMPO, 2,2,6,6-tetramethylpiperidine-1-oxyl; THRβ, thyroid hormone receptor beta; TIMP1, tissue inhibitor of metalloproteinases 1; TNF-α, tumor necrosis factor alpha; TRPML1, transient receptor potential mucolipin 1; Tregs, regulatory T cells; UPR, unfolded protein response; UUO, unilateral ureteral obstruction; VDAC1, voltage-dependent anion channel 1; VEGF-A, vascular endothelial growth factor A; WNT, Wnt/β-catenin signaling pathway†; XBP1s, spliced X-box binding protein 1; YAP, Yes-associated protein; YTHDF3, YTH N6-methyladenosine RNA binding protein 3; 18β-GA, 18β-glycyrrhetinic acid; 4-PBA, 4-phenylbutyric acid; 5-AZA-dC, 5-aza-2′-deoxycytidine.
